# IgE sensitization to inhalant allergens and the risk of airway infection and disease: A population-based study

**DOI:** 10.1371/journal.pone.0171525

**Published:** 2017-02-09

**Authors:** Tea Skaaby, Lise Lotte Nystrup Husemoen, Betina Heinsbæk Thuesen, Runa Vavia Fenger, Allan Linneberg

**Affiliations:** 1 Research Centre for Prevention and Health, Capital region of Denmark, Glostrup, Denmark; 2 Department of Clinical Experimental Research, Glostrup University Hospital, Glostrup, Denmark; 3 Department of Clinical Medicine, Faculty of Health and Medical Sciences, University of Copenhagen, Copenhagen, Denmark; Forschungszentrum Borstel Leibniz-Zentrum fur Medizin und Biowissenschaften, GERMANY

## Abstract

**Background:**

Immunoglobulin E (IgE) sensitization, which is the propensity to develop IgE antibodies against common environmental allergens, is associated with a lymphocyte T-helper type 2 (Th2) skewed immune response and a high risk of allergic respiratory disease. Little is known about whether IgE sensitization confers an increased risk of respiratory infections in adults. We investigated the association between IgE sensitization and the incidence of acute airway infections, other infections and chronic lower airway disease events as recorded in nation-wide registries.

**Methods:**

We included 14,849 persons from five population-based studies with measurements of serum specific IgE positivity against inhalant allergens. Participants were followed by linkage to Danish national registries (median follow-up time 11.3 years). The study-specific relative risks were estimated by Cox regression analysis, meta-analysed, and expressed as hazard ratios, HRs (95% confidence intervals, CIs).

**Results:**

The relative risks for IgE sensitized vs. non-sensitized were: for pneumonia (HR = 1.20, 95% CI: 1.01, 1.41), other acute airway infection (HR = 0.86, 95% CI: 0.60, 1.22), infection (HR = 1.06, 95% CI: 0.90, 1.24), asthma (HR = 2.26, 95% CI: 1.79, 2.86), and other chronic lower airway disease (HR = 1.31, 95% CI: 1.08, 1.58). In never smokers, the higher risk of pneumonia (HR = 1.73, 95% CI: 1.23, 2.44) and asthma (HR = 3.17, 95% CI: 2.10, 4.76) among IgE sensitized was more pronounced.

**Conclusions:**

IgE sensitization was associated with a higher risk of asthma, other chronic lower airway diseases, and pneumonia. However, the association between IgE sensitization and pneumonia may be explained by undiagnosed asthma causing the pneumonia. Further studies are needed for confirmation.

## Introduction

Allergic respiratory diseases have increased markedly in urbanized, westernized, and affluent populations [1,2]. Immunoglobulin E (IgE) sensitization refers to the production of allergen-specific IgE. IgE sensitization to an allergen is not the same as being allergic to the allergen. Individuals may produce IgE to allergens without developing symptoms if exposed to the allergen, i.e. individuals may or may not have allergic symptoms. It is unclear why some individuals demonstrate only sensitization while others have active allergic disease. IgE sensitization is associated with a lymphocyte T-helper type 2 (Th2) polarization, favouring Th2-derived cytokine production and production of allergen-specific immunoglobulin E (IgE) antibodies and a reduced lymphocyte T-helper type 1 (Th1) response [3]. Hence, it has been hypothesised that the reduced Th1 response could lead to a higher risk of infection [3].

Previous studies have suggested that persons with IgE sensitization and atopic disease may have a higher susceptibility to infection [3–6]. Since IgE sensitization has been shown to be inversely associated with smoking [7,8], and smoking may be associated with a higher risk of pneumonia and other respiratory diseases, smoking habits may be a (negative) confounder of the association between IgE sensitization and pneumonia. Furthermore, in epidemiological studies it is often difficult to discriminate between asthma and chronic obstructive pulmonary disease (COPD), the latter having a well-known high risk of hospitalization with pneumonia and exacerbation of disease. Hence, to avoid misclassification of COPD as asthma and confounding by smoking we performed additional analyses stratified by smoking status and expected any possible effects of IgE sensitization on infection and disease risk to be more evident in never smokers.

To our knowledge, only one relatively small previous study has investigated the prospective association between an objective marker of sensitization and risk of respiratory infections in adults [5]. Allergic respiratory disease is often underdiagnosed and the risk associated with objective biomarkers of atopic disease may add knowledge about the potential burden of atopic disease. IgE sensitization is sometimes asymptomatic, although it is associated with lower airway inflammation. We investigated the association between IgE sensitization, defined as specific IgE positivity against inhalant allergens, and the incidence of acute airway infection, infection and chronic lower airway disease in five Danish population-based cohorts.

## Materials and methods

### Ethics

Participants gave their informed written consent, each of the studies was approved by the Ethics Committee of Copenhagen and the Danish Data Protection Agency, and we followed the recommendations of the Declaration of Helsinki as previously stated [9,10].

### Study populations

We used the following five population based studies: Monica1, Inter99, Health2006, the 1936-cohort, and the Allergy98 study. They were recruited from the Danish Central Personal Register as random samples of the population in the southern part of Copenhagen and included questionnaires, physical examinations, and blood tests as previously described in detail [9,10]. An overview of the studies has previously been published [11].

The cohorts have been described in detail previously [9,10], but to recapture this information: A sample of 7,931 persons 18–69 years of age was invited to a general health examination in the Health2006 study (14). A total of 3,471 (43.8%) persons were examined from 2006–2008. In The Monica1 study, 4,807 persons with Danish citizenship at the ages 30, 40, 50 and 60 years old were invited (1982–84). The participation rate was 79%, yielding a total of 3,785 participants (12). In the Inter99 study (1999–2001), 12,934 persons from 30–60 years of age were invited. A total of 6,784 persons participated, and the participation rate was thus 52.5% (13). The study was a population-based randomized controlled trial (CT00289237, ClinicalTrials.gov), and it investigated the effects of lifestyle intervention on cardiovascular disease (13). The Copenhagen Allergy study that was initiated in 1990 included a group of persons randomly selected from the general population and a selected group of persons with allergic respiratory symptoms (that was recruited from a random sample of the general population by a screening questionnaire). We used data from the follow-up health examination in 1997–1998 (that is referred to as ‘Allergy98’) where 1,966 persons aged 15–77 years with Danish citizenship were invited. A total of 1,216 persons (61.9%) participated (15). And finally, the 1936-cohort was initiated in 1976 where a total of 1,200 randomly selected persons born in 1936 (40 years of age at the time of the study) were invited for a health examination focusing on cardiovascular disease risk factors. Between 1976 and 1977, 1,052 persons were examined (participation rate 87.7%). The 1936 cohort has previously been described (16;17).

**Fig 1:**
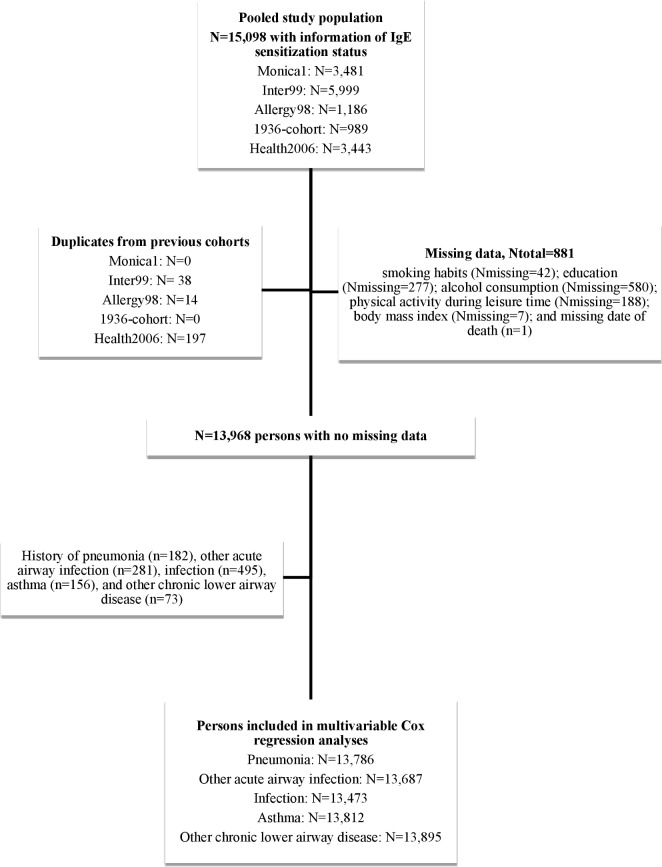
Flowchart of the included study populations.

From these five studies, 15,098 individuals had measurements of serum specific IgE, and of these, a total of 249 persons participated in more than one of the studies, and these persons were included only in the study in which they were first examined as described in detail previously [9,10]. We thus included a total of 14,849 persons (Inter99: 5,961; Allergy98: 1,172; 1936-cohort: 989; Health2006: 3,246; Monica1: 3,481) (Fig 1).

### Assessment of IgE sensitization

The following information on assessment of IgE sensitization has previously been described in detail [9,10]. In both the 1936-cohort and the Monica1 study, serum specific IgE positivity measurements were performed by the ADVIA Centaur Allergy Screen assay (Bayer HealthCare Diagnostics division, Tarrytown, N.Y., USA) (18). This is a multi-allergen assay for the detection of specific serum IgE antibodies to 19 common inhalant allergens. IgE sensitization was defined as a positive result in accordance with instructions from the manufacturer.

The ADVIA Centaur sIgE assay (Bayer Corporation, New York, NY) was used for measurements of serum specific IgE to mite (Dermatophagoides [D.] pteronyssinus), cat, grass and birch in the Allergy98 and the Health2006 studies (In the Allergy98 study in addition dog and mugwort) (19). The IMMULITE 2000 Allergy Immunoassay System was used for measurements of specific IgE to mite (D. pteronyssinus), cat, grass, and birch in the Inter99 study (20). In the Allergy98, the Health2006, and the Inter99 studies, the analysis for specific IgE was positive if the measurements were ≥0.35 kU/l. IgE sensitization was defined as one or more positive tests for specific IgE against the allergens tested.

### Registry-based diagnoses

The permanent and unique personal civil registration number of every person who lives in Denmark enables linkage of data from national registers on an individual level. Information on diseases according to the *International Classification of Diseases* (ICD) was obtained from the Danish National Patient Register [12]. The register holds information on all admissions to Danish hospitals since 1978. Outpatient visits such as emergency department visits have been recorded in the registries since 1995 and are included in the analyses. The Danish Registry of Causes of Death provided diagnoses suspected to be the cause of death [13]. Fatal and non-fatal events were combined. The included diagnoses are summarized in Table 1.

**Table 1:** Categorization of acute airway infection, infection, and chronic lower airway disease according to ICD-8 and ICD-10 codes.

	ICD-8	ICD-10
Pneumonia	Pneumonia (480–486)	Pneumonia (J12–J18)
Other acute airway infection	Acute respiratory infections, except influenza (460–466), Influenza (470–474)	Acute upper respiratory infections (J00–J06), Influenza (J09–J11), Other acute lower respiratory infections (J20–J22)
Infection	Certain infectious and parasitic diseases (0–136)	Certain infectious and parasitic diseases (A, B)
Asthma	Asthma (493)	Asthma (J45), Status asthmaticus (J46)
Other chronic lower airway disease	Bronchitis (490), Chronic bronchitis (491), Emphysema (492)	Bronchitis (J40), Simple and mucopurulent chronic bronchitis (J41), unspecified chronic bronchitis (J42), Emphysema (J43), Other chronic obstructive pulmonary disease (J44), Bronchiectasis (J47)

Abbreviations: ICD, International Classification of Disease.

Participants with a registry-based diagnosis of the event before baseline were excluded from the prospective analyses (Fig 1). The Danish Civil Registration System provided information on death and emigration status [14]. We followed the participants until 31 December 2010.

### Other variables

As previously described in detail [9,10], the questionnaires provided information on the following variables: physical activity during leisure time (sedentary, light, or moderate/vigorous); education/vocational training (no education beyond basic education [basic education includes primary and lower secondary education for 9 or 10 years], education including students); alcohol consumption (drinks per week); and smoking habits (never smokers; former smokers; light smokers <15 g/day; moderate smokers 15-<25 g/day; heavy smokers ≥25 g/day). Body mass index (BMI) was calculated as weight divided by height squared.

The number of missing covariate values was: sex (N_missing_ = 0); education (N_missing_ = 277); physical activity during leisure time (N_missing_ = 188); smoking habits (N_missing_ = 42); alcohol consumption (N_missing_ = 580); BMI (N_missing_ = 7); and missing date of death (n = 1) giving a total of 881 persons (5.9%) with a missing value in at least one of the variables (Fig 1).

### Statistical analyses

The analyses were performed with SAS, version 9.4 (SAS Institute Inc Cary, NC USA). The P-values were two-sided, and the p-values<0.05 were defined as statistically significant. Multivariate Cox regression analyses were used to determine the association of IgE sensitization and the incidence of pneumonia, other acute airway infection, infection, asthma, and other chronic lower airway disease. Age was used as underlying time axis (thus age is automatically adjusted for), and we used delayed entry. Persons experiencing an event contributed risk time until the time of the event. The few persons lost to follow-up contributed risk time until the time of their last registered activity. The estimates are presented as hazard ratios, HR (95% confidence intervals, CI). We only included persons with complete information on all considered variables, and we adjusted for gender, study population, education, physical activity, smoking habits, alcohol intake, and BMI.

We used Stata, version 12.1 (StataCorp LP, College Station, Texas, USA) to perform meta-analyses [15] of the study specific estimates by the inverse variance method in fixed effects models. We expected the associations to be similar across the studies because the study populations are comparable and from the same area. There was no sign of substantial heterogeneity across studies as assessed by the I^2^-test (all I^2^<50%).

Also, there were no statistically significant interaction between IgE sensitization and smoking habits, except for the outcomes asthma and other chronic lower airway disease. To examine the effects of misclassification of asthma with COPD and pneumonia with exacerbation of COPD, we also stratified the analyses by smoking habits.

In additional multivariable analyses, we excluded persons with self-reported asthma at baseline (variable only present in Health2006, Inter99 and Allergy98) and a registry-based diagnosis of asthma before and during follow-up, respectively. We also performed these analyses restricted to never smokers. To explore a possible effect of undiagnosed asthma at the time of pneumonia, we analyzed the association between IgE sensitization and”pneumonia without asthma” by logistic regression analysis. Persons with a diagnosis of asthma or pneumonia at baseline were excluded.”Pneumonia without asthma” was defined as a diagnosis of pneumonia among persons without a diagnosis of asthma during follow-up.

## Results

Table 2 shows the baseline characteristics of the participants according to study population and sensitization status expressed as mean (standard deviation, SD) or % (number). The IgE sensitization frequency ranged from 14.9–37.3% in the five study populations. IgE sensitization was associated with male sex, young age, higher consumption of alcoholic drinks, higher BMI, and vocational training.

**Table 2:** Baseline characteristics according to study population and sensitization status.

	Monica1 Mean (SD)/% (n)	Allergy98 Mean (SD)/% (n)	Inter99 Mean (SD)/% (n)	Health2006 Mean (SD)/% (n)	1936-cohort Mean (SD)/% (n)	Non-sensitized Mean (SD)/% (n)	Sensitized Mean (SD)/% (n)	P-value*
**Gender**								
Male	50.6 (1761)	45.7 (535)	49.2 (2930)	45.2 (1467)	46.7 (462)	46.1 (5006)	53.8 (2149)	<0.0001
Female	49.4 (1720)	54.3 (637)	50.8 (3031)	54.8 (1779)	53.3 (527)	53.9 (5849)	46.2 (1845)	
**Age** (years)	45.0 (11.1)	40.0 (15.1)	46.1 (7.9)	49.0 (13.1)	40.4 (0.4)	46.3 (10.9)	43.7 (10.4)	*<0*.*0001*
**Education**								
No	29.9 (1041)	28.8 (336)	16.8 (962)	13.8 (440)	28.5 (282)	22.3 (2380)	17.4 (681)	<0.0001
Yes	70.1 (2440)	71.2 (832)	83.2 (4777)	86.2 (2756)	71.5 (706)	77.7 (8276)	82.6 (3235)	
**Alcohol** (drinks/week)	8.9 (11.5)	6.8 (8.0)	10.4 (13.5)	9.7 (10.3)	8.6 (11.2)	9.3 (11.9)	10.0 (11.9)	*0*.*0003*
**BMI** (kg/m^2^)	24.6 (3.9)	25.7 (4.6)	26.3 (4.5)	25.9 (4.7)	23.9 (3.7)	25.5 (4.4)	25.8 (4.5)	*<0*.*0001*
**Smoking habits**								
Never smokers	25.7 (895)	42.3 (492)	35.0 (2087)	41.9 (1345)	32.8 (324)	32.7 (3545)	40.2 (1598)	<0.0001
Former smokers	18.5 (644)	17.8 (207)	25.3 (1505)	32.2 (1035)	13.3 (131)	24.2 (2621)	22.6 (901)	
Light smokers	25.3 (882)	18.9 (219)	15.3 (911)	12.2 (392)	21.7 (215)	18.3 (1984)	16.0 (635)	
Moderate smokers	24.8 (863)	15.1 (175)	17.8 (1060)	10.4 (333)	26.8 (265)	19.0 (2056)	16.1 (640)	
Heavy smokers	5.7 (197)	5.9 (69)	6.7 (398)	3.4 (109)	5.5 (54)	5.8 (623)	5.1 (204)	
**Physical activity**								
Sedentary	28.3 (985)	26.0 (303)	21.6 (1257)	18.5 (595)	34.7 (343)	24.1 (2585)	22.8 (898)	0.0026
Light	51.4 (1787)	50.3 (587)	61.5 (3581)	60.4 (1938)	51.1 (505)	57.6 (6179)	56.4 (2219)	
Moderate/vigorous	20.3 (705)	23.7 (277)	16.9 (981)	21.1 (676)	14.3 (141)	18.3 (1963)	20.8 (817)	
IgE sensitization***	17.1 (594)	37.3 (437)	34.5 (2058)	23.4 (758)	14.9 (147)	NA	NA	NA

* Chi-square or *Kruskal-Wallis test*.

*** Serum specific IgE positivity to inhalant allergens.

Abbreviations: BMI, body mass index; SD, standard deviation; NA, not applicable; BMI, body mass index.The baseline characteristics according to IgE sensitization status were compared with the Chi-test (categorical variables) or the Kruskal-Wallis test (continuous variables).

**Fig 2:**
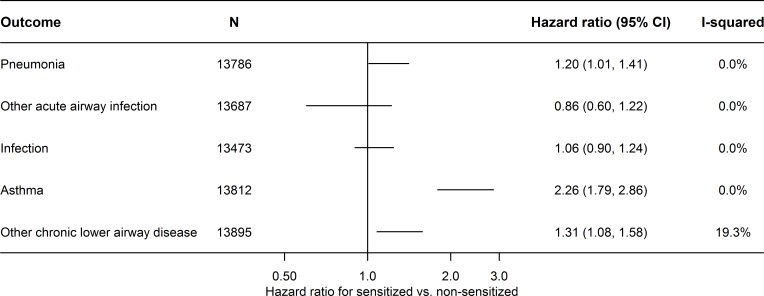
Fixed effects meta-analysis of the association between IgE sensitization and disease. Abbreviations: CI, confidence interval; HR, hazard ratio.

The results of the meta-analyses of the study-specific estimates from Cox regression analysis showed that IgE sensitization was associated with a higher risk of pneumonia, asthma and chronic lower airway disease (Fig 2).

The median follow-up was 11.3 years (Inter99: 11.0; Allergy98: 12.6; 1936-cohort: 33.8–33.9; Health2006: 3.5; Monica1: 27.3–27.4 years). The number of events was for pneumonia: N = 918, other acute airway disease: N = 216, infection: N = 956, asthma: N = 329, and other chronic lower airway disease: N = 713.

**Fig 3:**
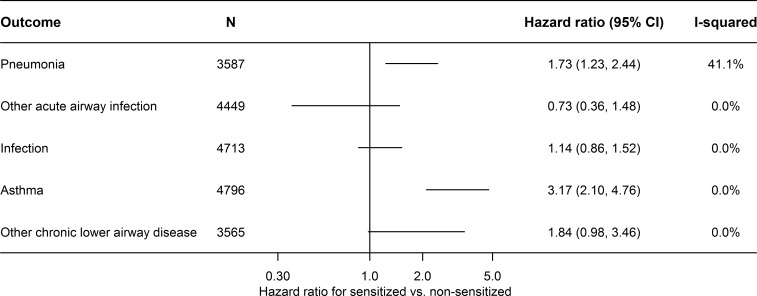
Fixed effects meta-analysis of the association between IgE sensitization and disease among never-smokers. Abbreviations: CI, confidence interval; HR, hazard ratio.

Fig 3 shows the associations of IgE sensitization with the included diseases for never smokers. In general, the analyses stratified by smoking status showed that never smokers compared to ever smokers had an increased risk of the included outcomes, except for risk of infection that was relatively unaffected by smoking habits (data not shown). In particular, IgE sensitized never smokers had a statistically significantly higher risk of pneumonia (HR = 1.73, 95% CI: 1.23, 2.44) and asthma (HR = 3.17, 95% CI: 2.10, 4.76) (Fig 3). However, there was also a higher risk of asthma among IgE sensitized former and light smokers, but no association between IgE sensitization and asthma among moderate and heavy smokers.

In additional analyses of the Health2006, Inter99 and the Allergy98 study, we excluded persons with self-reported asthma at baseline (including 8445 persons and a total of 214 events), and this yielded a HR of 1.12 (95% CI: 0.83, 1.51) of pneumonia for IgE sensitized vs. non-sensitized and restricting these analyses to only include never smokers (3241 persons and 57 events) yielded a HR of 1.20 (95% CI: 0.69, 2.08). In further analyses with exclusion of persons with a registry-based diagnosis of asthma before or during follow-up (including 13331 persons and 829 events), we found a HR of 1.12 (95% CI: 0.93, 1.33) of pneumonia for IgE sensitized vs. non-sensitized, and restricting these analyses to only include never smokers (4643 persons and 165 events) yielded a HR of 1.53 (95% CI: 1.08, 2.19). The estimates were largely similar when we only included primary diagnoses and inpatient contacts and excluded deaths (data not shown). Regarding mortality, we found that death caused by pneumonia for sensitized vs. non-sensitized had an adjusted HR = 0.91 (95% CI: 0.60, 1.40; p = 0.678). The odds ratio for pneumonia without asthma during follow-up was 2.78 (95% CI: 1.47, 5.23, p = 0.002) for IgE sensitized vs. non-sensitized.

## Discussion

In a large population-based study using registry-based follow-up to obtain information on respiratory infections and diseases, we found that IgE sensitization was associated with a significantly higher risk of a registry-recorded event of pneumonia, asthma and other chronic lower airway diseases. The higher risk of asthma associated with IgE sensitization is well-known, while the observed increased risk of pneumonia is not generally recognized as a co-morbidity of IgE sensitization. We found no association between IgE sensitization and infections in general.

Our results are in line with a study by Jung et al who found that atopic disease other than asthma was associated with an increased risk of serious pneumococcal disease [6]. Cirillo et al found that young adult patients with allergic rhinitis had more numerous and prolonged respiratory infections than non-allergic persons, and Rantala et al reported that atopic adults had more respiratory infections than non-atopics [5] and the number of respiratory infections increased with increasing levels of specific IgE antibodies to common allergens [3]. Ciprandi et al found that allergic children with a positive skin prick test or a history of allergic disease had more and more severe respiratory infections than non-allergic children [4]. Regarding atopy and chronic airway disease, Fattahi et al found that atopy is a risk factor for respiratory symptoms in COPD patients [16]. The development of immune tolerance with allergen-specific immunotherapy has long been successfully used in the treatment of IgE mediated allergic diseases. Of note, Ciprandi et al found that 40 children treated with allergen-specific immunotherapy had less severe extra-allergic surrogate markers of infection in comparison with controls [17]. In another study, Ciprandi et al found that sublingual immunotherapy may be able to reduce the number and duration of upper respiratory infections in patients with allergic rhinitis [18].

The potential mechanisms that may underlie the positive associations between IgE sensitization and pneumonia are unclear. It may involve both the suggested reduced Th1 response among IgE sensitized that may be important to the protection against infections as well as the mucosal inflammation and decreased defence against infectious agents associated with allergic diseases in general, e.g. among patients with atopic dermatitis [3]. Other possible mechanisms for the observed association between IgE sensitization and infection include a deficient interferon response; impaired alveolar macrophage function; changes of airway epithelium which may increase the susceptibility to infection; and genetic effects since many genes that are associated with the susceptibility to e.g., atopic disease are functional immune response genes [19]. In addition, several common external factors are associated with both increased susceptibility to infection and with asthma exacerbations or asthma control, e.g., smoking [19]. Of note, however, another study by Rantala et al, showed that recent respiratory infections were strong determinants for adult-onset asthma [20]. Other previous studies have found more frequent and more severe infections such as pneumonia in persons with atopy and asthma, both among children and adults [19,21–23]. This suggests that IgE sensitization may not be causally related to the susceptibility to infections but rather that IgE sensitization and infection share common pathways, or that persons more prone to infection develop atopic disease more frequently while keeping their propensity to get infections. However, we excluded persons with a history of the disease of interest prior to the time of assessment of IgE sensitization thereby minimizing the risk of reverse causation, i.e. infection/airway disease having an influence on IgE sensitization status. Regarding chronic lower airway diseases, the observed significantly higher risk of registry-recorded events among IgE sensitized, it is possible that, in persons with lower chronic airway disease, IgE sensitization does confer an increased risk of hospital admission due to exacerbation of disease. Another explanation could be misclassification of asthma as chronic lower airway disease. In some patients, there may be overlap between disease phenotypes or it may be difficult to discriminate between different diagnoses.

The strengths of our study include the large population-based samples and the longitudinal design which is superior to cross-sectional ones to demonstrate temporal relationships. The main analyses in the current study are Cox regression analyses since our outcome variable of interest is the time to event where the hazard ratio can be roughly interpreted as the incidence rate ratio [24]. Cox regression analysis works for censored survival times and suits our study with long-term and variable length follow-up, where a considerable number of persons whose observed times are censored before the end of the study which is not well taken care of by e.g., logistic regression analysis where these persons must be omitted or assumed not to have had the event. Also, we used standardised registry-based diagnoses and a long-term follow-up with few persons lost. Another strength is the use of measurements of serum specific IgE positivity to inhalant allergens. Commercially available immunoassays were used to determine serum specific IgE. We have previously reported that these results are in agreement with skin prick test reactivity and allergic respiratory disease [25,26]. However, it should be borne in mind that IgE sensitization does not reflect allergic respiratory disease per se. The use of three different methods to determine serum specific IgE positivity could potentially bias the results. However, there were no signs of significant heterogeneity between the studies.

The limitations of the study include the relatively low participation in some of the cohorts and the fact that we did not include information on self-reported diagnosis of allergy and asthma in the main analyses. We only had information on self-reported asthma diagnosis in three of the cohorts. However, the results from additional analyses with exclusion of persons with self-reported asthma diagnosis at baseline or a registry-based diagnosis of asthma either before or during follow up were in line with the main results, although the results from the additional analyses were only statistically significant when we included never smokers only. Also, as we only included cases where persons died, were seen in hospital either as inpatient, outpatient or emergency room contacts, we likely missed a substantial part, i.e. because most cases are probably treated by their private practitioner. However, we included the most severe cases, i.e. those that were hospitalized. There were no reported cases of the diagnoses of interest before 1976 for the 1936-cohort, probably because reporting to the register was incomplete. Consequently, there may be some misclassification of some of the persons (classified as incident cases in the 1936-cohort) that should have been classified as having a history of the event and excluded from the Cox regression analyses, had the register been more complete before 1976. It is also a potential limitation that we used data from five different studies, as this may induce biases. However, the meta-analyses showed no sign of heterogeneity across studies. In addition, we did not exclude immunocompromised subjects which may confound the finding if the immunocompromised were unequally distributed in sensitized and non-sensitized. However, the very low prevalence in the general population makes this a minor risk. It may be discussed whether reporting of incidence and the use of Cox regression analysis are appropriate since it only considers the first incident of, e.g., pneumonia, while failing to notice a subsequent diagnosis of, e.g., asthma. Indeed, there is a risk of misclassification of the outcome since pneumonia may be a sign of undiagnosed asthma, and this may explain some of the association between IgE sensitization and pneumonia. Although we used both self-reported and registry-based diagnosis of asthma, underreporting of asthma may still be an issue, since it may be missed when treating the exacerbations only. The exclusion of persons with a known asthma diagnosis at baseline and stratification by smoking habits may not entirely solve this problem.

The high and increasing prevalence of allergic disease warrants studies that investigate possible health effects of IgE sensitization beyond allergic diseases. Previously, we have found that IgE sensitization was not associated with diseases such as autoimmune disease or cancer [9,10,27,28]. In the current study we found that IgE sensitization was associated with a significantly higher risk of asthma and other chronic lower airway diseases as expected but also with a significantly higher risk of registry-recorded events of pneumonia. However, the positive association between IgE sensitization and pneumonia may be explained by an undiagnosed asthma causing the pneumonia, and further studies are needed for confirmation.
